# Long‐Term Glycemic Control and Pharmacotherapy in Type 2 Diabetes Mellitus: A Descriptive Analysis of 10‐Year Trends in a Statewide Sample

**DOI:** 10.1155/jdr/3531705

**Published:** 2026-06-07

**Authors:** Helen N. Chen, Michael Weiner, Julian Wolfson, Tamara Hannon, Patrick Balius, Titus Schleyer

**Affiliations:** ^1^ Division of Nursing Science, School of Nursing, Rutgers University, New Brunswick, New Jersey, USA, rutgers.edu; ^2^ Department of Family Medicine and Community Health, Rutgers Robert Wood Johnson Medical School, New Brunswick, New Jersey, USA, rutgers.edu; ^3^ Center for Biomedical Informatics, Regenstrief Institute, Inc., Indianapolis, Indiana, USA, regenstrief.org; ^4^ Department of Epidemiology, Richard M. Fairbanks School of Public Health, Indiana University, Indianapolis, Indiana, USA, indiana.edu; ^5^ Center for the Study of Healthcare Innovation, Implementation and Policy, VA Greater Los Angeles Healthcare System, U.S. Department of Veterans Affairs, Los Angeles, California, USA, va.gov; ^6^ Division of General Internal Medicine, Department of Medicine, David Geffen School of Medicine at UCLA, Los Angeles, California, USA, ucla.edu; ^7^ Division of Biostatistics & Health Data Science, School of Public Health, University of Minnesota, Minneapolis, Minnesota, USA, umn.edu; ^8^ Department of Pediatrics, School of Medicine, Indiana University, Indianapolis, Indiana, USA, indiana.edu; ^9^ Division of Environmental Health Sciences, School of Public Health, University of Minnesota, Minneapolis, Minnesota, USA, umn.edu; ^10^ Division of General Internal Medicine and Geriatrics, Department of Medicine, School of Medicine, Indiana University, Indianapolis, Indiana, USA, indiana.edu

**Keywords:** glycemic control, longitudinal, population health, Type 2 diabetes

## Abstract

**Background:**

Little is known about prescribing and glycemic control trends for Type 2 diabetes (T2D) drug classes across populations.

**Objective:**

The aim of this study is to assess long‐term glycemic control in a statewide sample prescribed with T2D drugs.

**Design:**

Descriptive analysis of retrospective 10‐year cohort data from the Indiana Health Information Exchange in the United States.

**Participants:**

Adults with T2D were prescribed at least one diabetes drug.

**Main Measures:**

Glycemic success is the number of A1c < 7% for age < 65 or <8% for age ≥65/(number of A1c tests) in any given year. Prescription rate is the number of subjects with a drug ordered in a year, divided by the population size in that year.

**>Results:**

Glycemic success was 60%, with year‐to‐year changes of 0%–1%. The prevalence of glycemic success was greater for females (mean of 62%–64%) than males (57%–59%); participants aged 65 or above (75%–89%) versus younger patients (43%–62%); and White (61%–63%) versus Black (51%–54%) patients. Biguanides (56%–63%) were most frequently prescribed, followed by sulfonylureas (29%–33%) and fast‐acting and long‐acting insulins (30%–32%). Black participants had more insulin prescriptions (47%–50%, vs. non‐Black, 9%–49%%). Newer drugs were more frequently prescribed for White than non‐White patients: 10%–17% versus 1.3%–17% for dipeptidyl peptidase 4 inhibitors; < 1%–9% versus 0.2%–8% for sodium/glucose cotransporter 2 inhibitors; and 5.3%–12% versus 1%–12% for glucagon‐like peptide receptor agonists. A greater number of diabetes drug classes ever prescribed was associated with lower glycemic success (1 class with 76%, vs. 2–3 classes with 55%, vs. 4–9 classes with 37%).

**Conclusions:**

Glycemic control did not substantively improve over a 10‐year period, despite the availability of new medications. Racial minorities received fewer prescriptions for newer drugs. Future research should examine factors contributing to prescription pattern differences and barriers to glycemic control.

## 1. Introduction

Diabetes is a common chronic disease, affecting more than 38 million Americans (about 1 in 10). Among people with diabetes, 90%–95% have Type 2 diabetes (T2D) [1]. Diabetes prevalence is expected to surge by nearly 70% by 2060 [2], and the proportion of uncontrolled diabetes, defined by A1c levels above 9%, rose from 13% to 16% from 2007 to 2014 [3]. Addressing this issue is crucial because long‐term control of A1c is instrumental in minimizing or preventing diabetes‐related organ damage in the eyes, kidneys, cardiovascular system, and nervous system [4]. Although efforts have been made to promote customized, patient‐centered care, considerable gaps persist between guideline recommendations and the quality of care in the United States, especially among marginalized patient groups [5]. Cost appears to be a significant driver in the gap between high‐quality care and achieving long‐term A1c control, as newer medications are not affordable for all. Emerging drugs have included glucagon‐like peptide receptor agonists (GLP‐1RA) in 2005, dipeptidyl peptidase 4 inhibitors (DPP‐4i) in 2006, and sodium/glucose cotransporter 2 inhibitors (SGLT‐2i) in 2013 [6]. Medications in these classes are nearly 200 times more expensive than older generic medications [7]. Furthermore, overall medication prices in the United States increased by nearly 58% from 2014 to 2019 [8].

Research comparing older medications like sulfonylureas to newer medications such as GLP‐1RA and SGLT‐2i indicates that the newer medications are more effective in lowering A1c levels and helping with weight control [9]. However, little is known about trends in prescribing new versus older drug classes and how prescriptions by drug class are distributed among various populations [7]. One study found that, during a median follow‐up of 8.3 years, less than half (45%) of participants were prescribed newer diabetes medications [6]. In that study, race and ethnicity were associated with initiation of newer medications, with Black and American Indian patients being less likely to be started on DPP‐4i, SGLT‐2i, and GLP‐1RA than White patients. To our knowledge, long‐term glycemic control success rates across race and drug classes are so far unreported. The purpose of our study was to assess long‐term glycemic control in a statewide sample of patients with prescriptions for T2D medications.

## 2. Methods

This longitudinal retrospective cohort study evaluated data from the Indiana Health Information Exchange [10], which comprises over 100 participating hospitals and outpatient practices, most belonging to one of five major health systems [11]. The health information exchange maintains data in the Indiana Network for Patient Care database [12]. This dataset contains longitudinal standardized data extracted from multiple sources including primary care clinics, specialty clinics, dialysis units, home care, and hospitals [10]. We identified a cohort of patients using Spratt et al.′s [13] T2D phenotype method based on International Classification of Diseases (ICD) codes, laboratory results, and prescribed T2D medications. Data were obtained for the period from January 1, 2009, to December 31, 2018. The relevant ICD‐9 codes were 250∗ (including 250.3 or 250.12), 249∗, 362∗ (diabetic retinopathy), and 357.2∗ (diabetic neuropathy). The ICD‐10 codes were E11, E12, and E13 (including their subcategories). The laboratory criteria were fasting plasma glucose > 126 mg/dL, 2‐h plasma glucose > 200 mg/dL during an oral glucose tolerance test, hemoglobin A1c > 6.5%, or random plasma glucose > 200 mg/dL. The 10 T2D drug classes were GLP‐1RA, amylinomimetics, sulfonylureas, meglitinide, DPP‐4i, SGLT‐2i, alpha‐glucosidase inhibitors, thiazolidinediones (TZD), biguanides, and insulin (rapid, long, short or regular, and intermediate‐acting). We also identified antidiabetic drug combinations. From the initial patient population of 531,995 with 1,868,101 year‐level observations, we excluded those without a diabetes drug prescription (*N* = 224,588), for a final cohort of 307,407 with a total of 876,656 year‐level observations from 2009 to 2018.

Glycemic target achievement was assessed using American Diabetes Association age‐based criteria: A1c ≤ 7.0% for those aged less than 65 years; A1c ≤ 8.0% for those aged 65 or more years [14]. These age‐differentiated targets reflect evidence‐based recommendations to individualize glycemic goals, particularly recognizing that older patients may benefit from less stringent targets to minimize hypoglycemia risk. Independent variables were gender, age group, race or ethnicity (White, Black, Asian, Pacific Islander, other or unknown race, and Hispanic), and prescription rate calculated from the number of subjects with a drug ordered in a year, divided by the population size in that year. The dependent variable was glycemic success. A person′s glycemic success was defined as the proportion of A1c test results meeting the target in any given year. The success of a group (e.g., females) was defined as the mean of the success of the individuals in the group. Medication cost was calculated for a 30‐day supply, considering the usual starting dose for noninsulin drugs, and a total daily dose of 30 units for insulin. Summarized at the year‐level, we computed for the 30‐day supply cost by averaging the unit costs of all medications listed within the same drug class in the US Department of Veterans Affairs national drug formulary [15]. Summary statistics, calculated with R software [16], were used to describe the trend of glycemic control by demographic characteristics and drug class prescribed.

## 3. Results

The sample′s yearly mean age ranged from 59 to 65, with slightly more female (51%–52%) and mostly White (73%–81%) representation; 1%–2% were Hispanic. Insulin was prescribed for 41%–46%. Overall, glycemic control success was 60%–62% with a yearly change of 0%–1%, whereas the prevalence of A1c > 8 increased from 25% to 27% over the 10 years. The fraction of study participants who were 65 or more years of age increased, reaching nearly half in the last 5 years (Table 1). Glycemic control success was 62%–64% among females versus 57%–59% in males; 75%–89% for age ≥65 versus 43%–62% for age 18–64; 61%–63% in White versus 51%–54% in Black; and 45%–48% in insulin users versus. 70%–73% in noninsulin users. Steadily for 10 years, about one‐third of the study population was prescribed two diabetes drug classes. The proportion of patients prescribed three or more drug classes remained stable at 23%–24% from 2010 to 2012, declined to 20% in 2013, then increased steadily from 25% in 2014 to 28% in 2018. After the 2013 dip, there was a sustained rise in polypharmacy (more than three drug classes) from 2014 onward. Similarly, the prevalence of A1c > 8 increased from 25% in 2009 to 27% in 2013, fluctuated between 25%–27% through 2017, and reached 28% in 2018. Notably, more than one in four patients had poor glycemic control (A1c > 8) despite prescribed pharmacologic treatment. The mean starting medication cost doubled from $57 to $118, and the median monthly cost increased from $6 to $34 over 10 years.

**Table 1:** Demographic, medical, and prescription characteristics of the study population from 2009 to 2018 (*N* = 876,656 year‐level observation from 308,407 patients).

	Year									
Characteristic	2009	2010	2011	2012	2013	2014	2015	2016	2017	2018
Patients (*n*)	53,401	63,545	75,285	78,816	93,121	88,649	89,674	99,361	104,171	130,724
Age (median)	59	60	61	62	63	65	64	65	65	65
Age group, years (%)										
18–44	16	14	13	13	11	11	11	10	10	10
45–54	23	22	20	20	18	16	16	16	16	16
55–65	30	29	29	28	28	26	26	26	26	27
65+	31	35	38	39	43	47	47	48	48	47
Female (%)	52	52	53	51	51	52	51	51	51	51
Race (%)										
White	73	74	76	77	80	80	81	82	81	79
Black	13	12	11	11	9	10	9	8	8	8
Asian	1	< 1	< 1	< 1	< 1	< 1	< 1	1	1	1
Pacific Islander	< 1	< 1	< 1	< 1	< 1	< 1	< 1	< 1	< 1	< 1
Native American	< 1	< 1	< 1	< 1	< 1	< 1	< 1	< 1	< 1	< 1
Other	12	12	11	11	10	8	8	8	9	11
Hispanic ethnicity (%)	1	1	1	1	1	2	1	1	1	1
Any insulin ever ordered (%)	41	41	44	46	44	45	46	44	44	43
A1c tests per year (mean)	2.0	2.1	2.2	2.2	2.2	2.0	2.0	2.1	2.1	2.1
A1c (mean)	7.4	7.3	7.4	7.4	7.4	7.5	7.5	7.4	7.4	7.5
Glycemic success: A1c met target (%)	60	61	61	60	60	60	60	62	61	60
A1c group (%)										
4.0–5.9	14	14	14	14	13	13	13	13	14	14
6.0–6.9	40	40	39	38	37	37	36	37	36	35
7.0–7.9	22	22	22	23	24	24	24	24	24	24
8.0–8.9	11	11	11	12	12	12	12	12	12	13
9.0–9.9	6	6	6	6	7	7	7	6	6	7
10+	8	7	7	8	8	8	8	7	8	8
Number of drug classes prescribed (%)										
1	41	43	42	43	48	41	41	41	40	39
2	34	34	34	34	32	34	33	32	32	32
3	17	16	16	16	14	17	17	18	18	18
4	6	5	6	6	5	6	7	7	7	8
5+	2	2	2	2	1	2	2	2	3	3
Estimated monthly drug cost in USD^**a**^										
Mean (IQR)	57 (42)	59 (42)	67 (43)	73 (43)	73 (44)	79 (44)	89 (49)	100 (209)	104 (210)	118 (288)
Median	6	6	11	12	11	12	24	34	34	34

*Note:* Prescribed is defined as “yes or no” that this drug class was prescribed during the follow‐up year.

^a^Cost for a 30‐day supply at the starting dose based on 2018 drug price from the Department of Veterans Affairs drug formulary.

**Figure 1:**
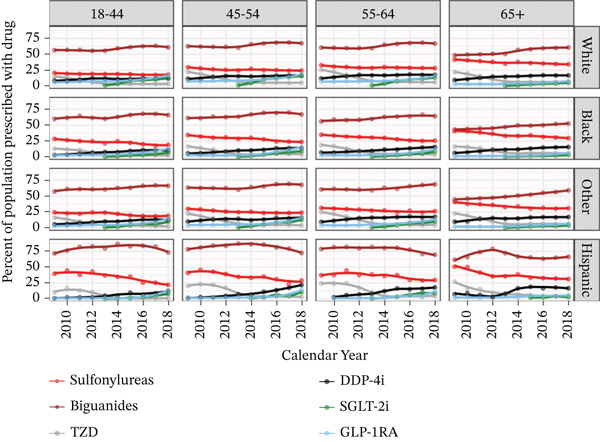
Oral drug and GLP‐1RA prescriptions for the study population, by age group and race or ethnicity from 2009 to 2018.

**Figure 2:**
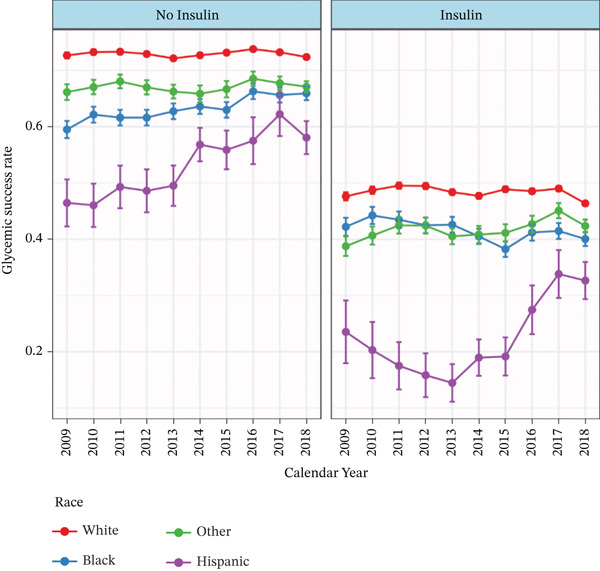
Glycemic control success, by insulin use and race or ethnicity, by year 2009–2018.

Biguanides were the most frequently prescribed drug class overall at 60% of the sample, followed by sulfonylureas (31%), rapid‐acting insulin (31%), and long‐acting insulin (30%). Patients prescribed a single drug class had the greatest glycemic success prevalence (Table 2). The median number of diabetes drug classes was two in all years. Lower rates of glycemic success occurred among those with higher numbers of drug classes ever prescribed within the 10‐year period (1 class with 76% vs. 2–3 classes with 45%–55% vs. 4–9 classes with 31%–37%). A quarter of prescriptions involved patients who were prescribed three or more drug classes (17%, 6%, and 2%; Table 2). These were mainly for the 18–64 age group (59%–66%), who had uncontrolled glycemic rates of 38%–50% and insulin prescription rates of 70%–95%. The prescription prevalence for newer drugs rose in 2014. However, these drugs were more frequently prescribed for White than non‐White patients: 0.6–1.2% versus 0.5%–0.8% for meglitinides, 10.3%–16.7% versus 1.3%–17.3% for DPP‐4i, 0.5%–9.3% versus 0.2%–8.0% for SGLT‐2i, and 5.3%–12.2% versus 1%–11.5% for GLP‐1RA (Figure 1). Approximately one‐third of those receiving costlier drugs prescriptions (DPP‐4i, GLP‐1RA, and SGLT‐2i) had A1c > 8 at 28%, 34%, and 38% (Table 2), and one‐third to one‐half were prescribed insulin. Black patients had higher insulin prescription rates (47%–50%) than other racial or ethnic group (9%–49%). For Black patients who were prescribed insulin, the glycemic success prevalence was lower as compared with White patients (38%–43% vs. 46%–50%) as shown in Figure 2.

**Table 2:** Patient characteristics and outcomes per person‐years by number of drug classes and age group 2009–2018.

Characteristics	Person‐years^a^ n (%)	Mean A1c (median)	Glycemic success: A1c met target (mean %)	Uncontrolled glycemic: *A*1*c* > 8 (mean %)	Estimated cost^b^ in USD mean (median)	Insulin prescribed *n* (%)	Non‐White *n* (%)
Age group							
18–44		7.9 (7.4)	42%	77%	80, 34	48,392 (48)	32,657 (32)
45–54	155,164 (18)	7.8 (7.3)	43%	71%	97, 17	67,049 (43)	41,653 (27)
55–64	238,420 (27)	7.5 (7.1)	48%	57%	99, 17	102,819 (43)	50,760 (21)
65+	382,175 (44)	7.1 (6.9)	80%	40%	76, 11	166,604 (44)	61,269 (16)
Number of drug classes							
1	366,186 (42)	6.9 (1.3)	76%	13%	26, 1	66,898 (18)	78,176 (21)
2	287,743 (33)	7.6 (1.8)	55%	29%	77, 35	148,618 (52)	62,895 (22)
3	147,347 (17)	8.0 (2.0)	45%	38%	158, 46	103,205 (70)	30,744 (21)
4	56,324 (6)	8.2 (2.0)	37%	45%	245, 300	48,121 (85)	11,130 (20)
5–9	19,050 (2)	8.5 (2.1)	31%	50%	358, 339	18,042 (95)	3,401 (18)
Drug class type							
Biguanide	525,187 (60)	7.3 (6.9)	63	23	74(2)	149,281 (28)	112,721 (21)
Insulin, rapid‐acting	268,807 (31)	7.9 (7.5)	48	37	96 (44)	268,807 (100)	56,254 (21)
Sulfonylureas	267,662 (31)	7.6 (7.3)	56	29	98 (6)	86,008 (32)	54,623 (20)
Insulin, long‐acting	261,872 (30)	8.2 (7.9)	40	45	112 (44)	261,872 (100)	56,108 (21)
DPP‐4i	126,534 (14)	7.6 (7.3)	55	28	338 (293)	43,640 (34)	22,972 (18)
TZD	77,422 (9)	7.4 (7.0)	61	23	112 (12)	23,776 (31)	15,881 (21)
GLP‐1RA	60,228 (7)	7.8 (7.5)	45	34	404 (332)	30,656 (51)	9448 (16)
Insulin, intermediate‐acting	52,101 (6)	8.2 (7.8)	43	43	61 (40)	52,101 (100)	17,363 (33)
SGLT‐2i	32,454 (4)	7.9 (7.7)	36	382	388 (497)	13,463 (41)	4693 (14)
Insulin, short‐acting (regular)	27,010 (3)	8.5 (8.2)	35	50	76 (46)	27,010 (100)	6713 (25)
Meglitinides	6595 (0.8)	7.3 (7.1)	64	23	165 (65)	2899 (44)	1026 (16)
Alpha‐glucosidase inhibitor	2522 (0.3)	7.6 (7.4)	56	30	142 (49)	993 (39)	604 (24)

*Note:* Data are based on 876,656 year‐level observations of 308,407 patients. Glycemic success percentages reflect year‐level observations, not monotherapy‐only patients. For each drug class, glycemic success was calculated among all person‐years in which that drug class was prescribed during that specific year, regardless of whether it was used alone or in combination with other medications. For example, for biguanide, glycemic success was 60%, calculated as 0.60 = 525,187/876,656 observation of person‐years. This represents the proportion of all person‐years in which biguanide was prescribed and the A1c target was met during that year. These person‐years include both biguanide monotherapy and biguanide used in combination with one or more additional drug classes. Thus, the reported percentages reflect real‐world glycemic outcomes during years in which a given drug class was prescribed, rather than outcomes limited to patients receiving that drug as monotherapy.

Abbreviations: DPP‐4i, dipeptidyl peptidase 4 inhibitors; TZD, thiazolidinediones; GLP‐1RA, peptide receptor agonists; SGLT‐2i, sodium/glucose cotransporter 2 inhibitors.

^a^Person‐years represent year‐level observations, with each row corresponding to one person in 1 year. Prescription orders were summarized within each year; thus, one person followed for 1 year equals one person‐year, and patients may contribute multiple person‐years during 2009–2018. The number and type of drug classes reflect prescriptions during that specific year. For example, among person‐years with one drug class prescribed, insulin was the sole agent in 18% of those years (0.18 = 66,898/366,186).

^b^Overall drug cost for a 30‐day supply at the starting dose based on 2018 drug price from the Department of Veterans Affairs drug formulary.

## 4. Discussion

This study analyzed glycemic control longitudinally across multiple healthcare settings using rich data from a large health information exchange. It examined yearly trends for various diabetes medication classes and population characteristics, unlike cross‐sectional studies that lack long‐term trend analysis. This study identified a persistent lack of improvement in glycemic control (glycemic success change was 0%–1%) for a large cohort of patients with T2D across a 10‐year period. The populations least likely to meet glycemic control targets were aged 18–64 (43%–62%), male (57%–59%), Black (51%–54%), and insulin users (45%–48%). Greater numbers of drug classes prescribed were insufficient to achieve glycemic control in this population (1 class with 76% vs. 2–3 classes with 45%–55% vs. four–9 classes with 31%–37%). Costlier drugs did not correspond to better glycemic outcomes (glycemic success rate ranged from 36% to 55% and uncontrolled glycemic rate from 28% to 38%). Our results suggest potential disparities in prescription patterns—not tested for statistical significance—with newer and more expensive drugs like DPP‐4i (22,972/126,534, 18%), GLP‐1RA (9,448/60,228, 16%), and SGLT‐2i (4,693/32,454, 14%) being prescribed less often for those in racial or ethnic minority groups.

Little improvement was seen in patients′ achieving their A1c target (remaining at 60%–62%). A possible explanation is that, after one drug class is insufficient, adding a second drug class may not be prescribed due to cost. Montvida et al.′s [17] comparison of two drug classes prescribed using a 20‐year (1995–2016) electronic health record dataset found that the adjusted probability of sustaining glycemic control over 2 years was higher in participants prescribed incretin or TZD (range 62%–75%), whereas insulin and sulfonylureas were associated with lower chances of sustainable control (range 54%–56%). One reason for a lack of progress in achieving glycemic control is therapeutic inertia: the failure to adjust treatment promptly when a patient′s target is not met. Therapeutic inertia often reflects an array of clinical, organizational, structural, societal, personal, financial, cultural, and environmental considerations. For instance, societal factors influence patients′ medication preferences, willingness to take drugs, and regularity in accessing diabetes care. Consequently, our data on prescription alone do not reveal the numerous factors underlying the prescribing decision. In another study, the median time to treatment intensification after an A1c exceeded the target was more than 1 year (range 0.3 to > 7.2 years) [18]. Excessive delays in intensifying treatment may result from inadequate monitoring [19]. Our study found that the mean A1c test frequency was two A1c tests per year, failing to meet the quarterly testing recommended by the American Diabetes Association [20]. Our finding is also consistent with prior studies showing that groups with poorer glycemic control (A1c > 7.5%) received fewer A1c tests [19, 21].

Certain subgroups demonstrated less successful glycemic control: adults <65 years of age, males, Black patients, and those prescribed with insulin. Overall, individuals aged ≥65 had better glycemic control, representing nearly half of both the 1‐drug and 2‐drug groups, with 55%–76% achieving their A1c targets. This finding is consistent with two studies using national samples that suggested that the older age group tends to perform well. In one study, those ≥60 were 75% more likely to achieve glycemic control [5]. In the other, compared with those aged 45–64, individuals ≥65 had higher odds (1.70), whereas those aged 18–44 had lower odds (0.53), of meeting the composite target for diabetes care such taking lowering cholesterol drug and meeting the A1c target [22]. Our study showed, Black patients prescribed with insulin therapy had a lower success rate for glycemic control. These results are consistent with others′ finding of lower likelihood of achieving the A1c target among Black patients [5] and non‐White individuals [22], Our result also aligns with the prior finding that Black patients had the highest prevalence of severe hyperglycemia (A1c > 10) of all racial/ethnic groups [23]. These findings highlight the potential influence of demographic factors on diabetes management and the need for tailored interventions and strategies to address disparities in glycemic control among specific subgroups, such as younger adults, males, and certain racial or ethnic populations, especially those requiring insulin therapy. These findings warrant further investigation with formal statistical analysis.

Our study found that those treated with insulin had less glycemic control compared with those who were never prescribed with insulin (Figure 2). Insulin prescriptions were high among patients who had ever been prescribed 3, 4, or 5–9 drug classes, at 70%, 85%, and 95%, respectively. The insulin increase trend in our sample is similar to the national trends for 2015–2016 [7] and 2016–2020 [24]. It is unknown whether this is due to a greater prevalence of persistent uncontrolled T2D requiring insulin or insufficient treatment intensification with newer drugs due to cost or other factors. The prevalence of glycemic success in our sample was trending steady around 50% for Whites versus 40% for Black and other races. Achieving and maintaining glycemic control is often difficult for insulin users [23, 25].

We were not surprised that, in our study population, among patients prescribed with a larger number of drug classes, the prevalence of glycemic success was lower, because patients requiring more medications often have more severe or complex cases of diabetes, making glycemic control more challenging to achieve. Over one‐third of patients ever prescribed 4 or 5–9 drug classes were ≥65 years of age (38% and 34%, respectively). These results may reflect clinicians′ concerns of treatment intensification, such as hypoglycemia. For patients ≥ 65, who are more than one‐third of those taking multiple diabetes medications, the high out‐of‐pocket costs associated with newer drugs like GLP‐1RA, SGLT‐2i, and DPP‐4i under Medicare Plan D may be a major deterrent to prescribing these medications and could contribute to therapeutic inertia. In 2019, the out‐of‐pocket cost for newer diabetes medications, such as GLP‐1RA, SGLT‐2i, and DPP‐4i, under Medicare Part D was 40–360 times higher than the cost of commonly covered sulfonylureas or TZD—exceeding $5000 annually [26]. We did not find other studies that assessed a link between diabetes drug costs and the prescribing pattern.

Previous studies have shown a delay in intensifying diabetes treatment, which is particularly pronounced for newer medications in the class of GLP‐1RA, SGLT‐2i, and DPP‐4i [27]. This treatment inertia is especially prevalent in racial and ethnic minority groups, where prescribing rates for these newer drugs tend to be lower [28]. Despite the introduction of newer drugs, we found that sulfonylureas and insulin prescription rates were essentially unchanged over the 10‐year period, as also found by Montvida et al. [27]. Their study reported that the rates of intensification with insulin and sulfonylureas did not decline, and the mean time to insulin initiation was marginally longer in the DPP‐4i group (7.1 years) and the GLP‐1RA group (6.6 years), compared with sulfonylureas (6.3 years). Our results were also consistent with Gopalan et al.′s findings that newer and more expensive drugs were prescribed less for patients in minority groups [28], contributing to therapeutic inertia with a delay in treatment intensification. Elhussein et al. [6] found that this problem of disparities in the initiation of newer drugs occurs even when adjusted for socioeconomic factors, particularly for GLP‐1RA.

A possible explanation for the delay in treatment intensification with newer drugs is the cost. In our study, the estimated mean monthly cost at starting dose was $118 in 2018. When a single drug class (a mean monthly cost of $26) becomes insufficient for a patient to reach A1c targets, treatment intensification can involve adding additional drug classes. Over the 10‐year period, the mean monthly costs escalated to $77 for 2 drugs, $158 for 3, $245 for 4, and $358 for 5–9 classes ever prescribed, an increase of $51–$332 compared with monotherapy (Table 2). Notably, between the market‐entry year of the first drug and December 2017, the costs of GLP‐1RA, DPP‐4i, and SGLT‐2i increased by approximately 10% annually [29]. Despite competition among manufacturers′ brands within the same class, having multiple drugs did not help lower the list price [29]. First‐line metformin has the lowest average per person lifetime cost ($92,000 vs. $135,000 for SGLT‐2i vs. $141,000 for GLP‐1RA) [30]. To be cost‐effective at $150,000 per quality‐adjusted life year, the costs would need to be under $5 per day for SGLT‐2i and under $6 per day for oral GLP‐1RA [30]. Insurance companies can use their negotiating power to secure lower prices from pharmaceutical manufacturers, so that lowering patients′ out‐of‐pocket costs can improve patient access to newer medications like SGLT‐2i and GLP‐1RA.

This study has limitations due to the lack of statistical testing for association or correlation. Without formal statistical testing, we cannot conclude that observed differences by race represent statistically significant disparities. Additionally, there was inconsistent information in our dataset on mixed‐race individuals, and the absence of insurance details. Individuals of mixed race answering “other” may have been excluded from one or more main racial categories, potentially affecting the distribution. The influence of insurance coverage and type on prescription orders cannot be ruled out, as certain prescriptions may depend on insurance formularies. Our study data may not generalize to other geographic areas, settings, or population groups. However, the study′s strength lies in its rich longitudinal data spanning 10 years, unlike many cross‐sectional studies that are limited to 5 years or less. Our data enabled a deeper assessment of trends, allowing observation of new drug introductions and the potential impact of marketplace insurance on prescriptions during the study period.

Further studies are needed to explore the mechanisms behind poor long‐term diabetes population health. Qualitative research should examine patient‐based factors such as access to care and adherence to prescriptions and recommended A1c testing, as well as clinician‐based factors such as patient outreach on returning to clinic for treatment adjustment, education about self‐management, and follow‐up of abnormal test results. Health system factors, such as using diabetes registries and specialized support staff, should also be investigated. Quantitative studies can assess variations in prescribing costlier drugs based on social determinants of health, insurance type, and access to care. Integrating qualitative and quantitative findings can provide a comprehensive understanding and inform strategies to improve glycemic control.

## 5. Conclusion

Over a 10‐year period, despite the introduction of new medications, the prevalence of glycemic success remained stagnant at 60% in our statewide cohort. This lack of improvement might be attributable to the under‐prescribing of newer drugs for treatment intensification due to cost concerns or other factors. Racial minorities received fewer prescriptions for higher‐priced drugs, though formal statistical testing would be needed to confirm the statistical significance of these observed differences. Future research should investigate the contributing factors behind these disparities and identify barriers hindering the achievement of glycemic control targets. Addressing these issues could improve disease management and outcomes for individuals with T2D.

## Funding

This study was supported by the U.S. National Library of Medicine (10.13039/100000092, T15LM012502); Advancing Translational Sciences Clinical and Translational Sciences (UL1TR002529).

## Disclosure

The authors take full responsibility for all the content of this publication.

## Ethics Statement

This work was approved by the Indiana University Institutional Review Board.

## Conflicts of Interest

Michael Weiner reports stock holdings in 3 M, Abbvie, Amgen, Bristol Myers Squibb, Centene, Conmed, Crispr Therapeutics, Dexcom, DXC Technology, Embecta, GE Healthcare Technologies, Globus Med, GSK, Haemonetics, Integer, Integra Lifesciences, Johnson & Johnson, Medtronic, Novo Nordisk, Nuvasive, Orthofix, Pfizer, Revvity, Roche, Stryker, Sonseonics, Teva Pharmaceutical Industries, Viaris, Veradigm, Walgreens, and Zimmer Biomet. The other authors declare no conflicts of interest.

## Data Availability

The data that support the findings of this study are available on request from the corresponding author. The data are not publicly available due to privacy or ethical restrictions.
